# Exploring the Potential of a Deep Learning Model for Early CT Detection of High-Grade Metastatic Epidural Spinal Cord Compression and Its Impact on Treatment Delays

**DOI:** 10.3390/cancers17132180

**Published:** 2025-06-28

**Authors:** James Thomas Patrick Decourcy Hallinan, Junran Wu, Changshuo Liu, Hien Anh Tran, Noah Tian Run Lim, Andrew Makmur, Wilson Ong, Shilin Wang, Ee Chin Teo, Yiong Huak Chan, Hwee Weng Dennis Hey, Leok-Lim Lau, Joseph Thambiah, Hee-Kit Wong, Gabriel Liu, Naresh Kumar, Beng Chin Ooi, Jiong Hao Jonathan Tan

**Affiliations:** 1 Department of Diagnostic Imaging, National University Hospital, National University Health System, Singapore 119074, Singapore; andrew_makmur@nuhs.edu.sg (A.M.); wilson.ong@mohh.com.sg (W.O.); ee_chin_teo@nuhs.edu.sg (E.C.T.); 2 Department of Diagnostic Radiology, Yong Loo Lin School of Medicine, National University of Singapore, Singapore 117597, Singapore; 3 Department of Computer Science, School of Computing, National University of Singapore, Singapore 117417, Singapore; junran@nus.edu.sg (J.W.); changshuo@u.nus.edu (C.L.); ooibc@comp.nus.edu.sg (B.C.O.); 4 Lee Kong Chian School of Medicine, Nanyang Technological University, Singapore 308232, Singapore; tran0104@e.ntu.edu.sg (H.A.T.); noah0005@e.ntu.edu.sg (N.T.R.L.); 5 Department of Orthopaedic Surgery, National University Hospital, National University Health System, Singapore 119074, Singapore; wsl.wangshilin@gmail.com (S.W.); doshhwd@nus.edu.sg (H.W.D.H.); doslll@nus.edu.sg (L.-L.L.); dosjst@nus.edu.sg (J.T.); doswhk@nus.edu.sg (H.-K.W.); gabriel_liu@nuhs.edu.sg (G.L.); dosksn@nus.edu.sg (N.K.); jonathan_jh_tan@nuhs.edu.sg (J.H.J.T.); 6 Biostatistics Unit, Yong Loo Lin School of Medicine, National University of Singapore, Singapore 117597, Singapore; medcyh@nus.edu.sg

**Keywords:** metastatic epidural spinal cord compression, spinal metastases, computed tomography, treatment delays, Bilsky grading scale

## Abstract

Background: Delay in diagnosing metastatic epidural spinal cord compression (MESCC) adversely impacts clinical outcomes. High-grade MESCC is frequently overlooked on routine staging CT scans. We aim to assess the potential of our deep learning model (DLM) in detecting high-grade MESCC and reducing diagnostic delays. Methods: This retrospective review analyzed 140 patients with surgically treated MESCC between C7 and L2 during 2015–2022. An experienced radiologist (serving as the reference standard), a consultant spine surgeon, and the DLM independently classified staging CT scans into high-grade MESCC or not. The findings were compared to original radiologist (OR) reports; inter-rater agreement was assessed. Diagnostic delay referred to the number of days elapsed from CT to diagnostic MRI scan. Results: Overall, 95/140 (67.8%) patients had preoperative CT scans. High-grade MESCC was identified in 84/95 (88.4%) of the scans by the radiologist (reference standard), but in only 32/95 (33.7%) of the preoperative scans reported by the OR. There was almost perfect agreement between the radiologist and the surgeon (kappa = 0.947, 95% CI = 0.893–1.000) (*p* < 0.001), and between the radiologist and the DLM (kappa = 0.891, 95% CI = 0.816–0.967) (*p* < 0.001). In contrast, inter-observer agreement between the OR and all other readers was slight (kappa range = 0.022–0.125). Diagnostic delay was potentially reduced by 20 ± 28 (range = 1–131) days. Conclusions: The original radiologist reports frequently missed high-grade MESCC in staging CT. Our DLM for CT diagnosis of high-grade MESCC showed almost perfect inter-rater agreement with two experienced reviewers. This study is the first to demonstrate that the DLM could help reduce diagnostic delays. Further prospective research is required to understand its precise role in improving the early diagnosis/treatment of MESCC.

## Simple Summary

Early diagnosis and treatment of metastatic epidural spinal cord compression (MESCC) are crucial in improving patient outcomes. We previously developed and externally validated a deep learning model for the automated detection of high-grade MESCC on computed tomography (CT) scans. The aim of our study is to assess the potential of this deep learning model in detecting high-grade MESCC on routine staging CT scans. We found that there was good inter-rater agreement between the deep learning model and two experienced reviewers in detecting high-grade MESCC, and that the deep learning model could also be effective in reducing diagnostic delays. Deep learning techniques show significant promise for advancing patient care through the expedient detection and diagnosis of MESCC, but further work should be carried out to fully elucidate their potential role and utility.

## 1. Introduction

Metastatic epidural spinal cord compression (MESCC) is estimated to occur in up to 5% to 10% of patients with cancer [1,2,3]. Delays in diagnosing and treating metastatic epidural spinal cord compression (MESCC) are associated with poorer surgical and functional outcomes in cancer patients [4,5]. While decompressive surgery and radiotherapy have been shown to be superior to radiotherapy alone for MESCC patients [6,7,8,9,10,11,12,13,14], the challenge lies in the timely identification of MESCC to select for prompt surgical treatment and improve patient outcomes [1]. Despite an increased number of surgically treated MESCC cases in recent years [15], delay in surgical treatment remains a significant predictor of poor postoperative outcomes and increased mortality [4,16,17,18,19,20].

Magnetic resonance imaging (MRI) is the gold-standard modality for diagnosing MESCC, which can be classified using the Bilsky scale; low-grade MESCC includes Bilsky grades 0 to 1b (thecal sac indentation without spinal cord contact), whereas high-grade disease (grades 1c to 3) involves progressively increased spinal cord compression [21]. Despite being the gold standard for diagnosing MESCC, the limitations of MRI, including cost and infeasibility for routine cancer follow-up, necessitate the exploration of alternative screening methods [22]. Staging computed tomography (CT), which is routinely performed in cancer follow-up, has emerged as an opportunity for earlier and opportunistic detection of MESCC when symptoms are less clear [23]. Earlier diagnosis of MESCC could facilitate the use of minimally invasive treatment modalities, such as external beam or stereotactic body radiotherapy [24], and improve treatment outcomes for patients.

Previous studies have shown that MESCC, diagnosed by MRI, is detectable in 80% of staging CT studies performed a month before the MRI [25]. However, MESCC diagnosis in staging CT scans is often missed by the original reporting radiologists, with a sensitivity of only 44% for general radiologists who typically lack expertise in interpreting MESCC and/or spinal MRI [23]. Deep learning models have recently been developed to aid in the interpretation of CT and MRI studies of the spine for metastases, including MESCC detection [26,27,28,29,30,31,32,33,34,35,36,37,38,39,40,41,42]. Most recently, Hallinan et al. (2023) further developed a deep learning model (DLM) for classifying MESCC on CT, which demonstrated high inter-rater agreement on their institutional data (κ = 0.872 on internal testing) and sustained high agreement in external testing (κ = 0.844) [43]. Although the predictions of the DLM were superior or comparable to those of both experienced and inexperienced radiologists, it remained uncertain whether earlier detection of MESCC on CT could reduce delays in the diagnosis and treatment of MESCC.

This study aims to compare the inter-rater agreement between a developed DLM [43], experienced reviewers, and original radiological reports in diagnosing high-grade MESCC on screening CT scans of the thorax, abdomen, and pelvis. The reference standard will be provided by an MRI performed post-CT, and the potential reduction in diagnostic delay will be measured as the number of days from the first CT showing high-grade MESCC to the diagnostic MRI.

## 2. Materials and Methods

### 2.1. Ethical Approval and Study Design

The study was conducted in accordance with the principles of the Declaration of Helsinki and approved by the Institutional Review Board of the National Healthcare Group, Singapore (approval number: 2022/00866; date of approval: 23 March 2023). A waiver for consent was obtained for this retrospective study, considering its minimal risk nature.

### 2.2. Patient Selection and Inclusion Criteria

This retrospective study included 140 patients who underwent surgical decompression and stabilization for MESCC between January 2015 and January 2022 at the National University Hospital, Singapore. The inclusion criteria were adult patients (>18 years old) with high-grade MESCC (Bilsky grades 2–3) between C7 and L2 on MRI and available CT scans of the thorax, abdomen, and pelvis (performed up to four months prior to the MRI). The exclusion criteria were primary vertebral tumors, prior surgeries, non-contrasted CT scans, and poor-quality images due to motion or scan-related artifacts.

### 2.3. Imaging Evaluation

**Figure 1:**
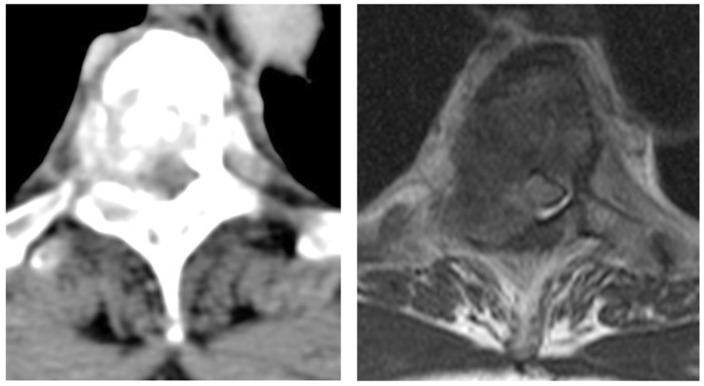
Example of high-grade (Bilsky grade 3) MESCC in a 43-year-old woman with breast cancer. Axial CT was performed in the portal-venous phase (**left**) with matching axial T2-weighted MRI (**right**) [43].

Transverse or axial CT images in the Digital Imaging and Communications in Medicine (DICOM) format (de-identified) were provided for MESCC assessment. These were reviewed by the readers using the institutional picture archiving and communication system (PACS) with various window and contrast settings available for optimized visual assessment. The CT scans were reviewed independently by a senior consultant musculoskeletal radiologist (J.T.P.D.H., with 12 years of experience) who served as the reference standard, and a consultant spine surgeon (J.H.J.T., with 6 years of experience). The scans were classified into two groups: those with high-grade MESCC and those without. None of the readers had access to the predictions of the deep learning algorithm. The experienced radiologist (reference standard) had access to the diagnostic MRI for comparison (Figure 1).

A deep learning model (DLM) that was previously developed and externally validated was then used to classify the CT scans using de-identified axial images in the abdominal window with a width and level of 400/50 (Hounsfield units). The predictions of the reader and DLM were also compared to the report by the original radiologist (OR) who interpreted the staging CT study.

### 2.4. Clinical Data Collection

**Figure 2:**
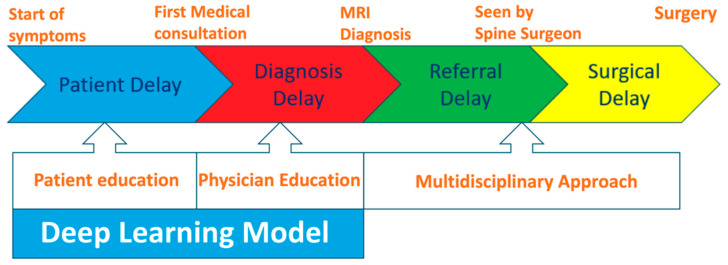
Potential delay intervals for patients with MESCC.

Clinical data collected for the patients with MESCC included baseline demographic characteristics (e.g., age, sex, race, Eastern Cooperative Oncology Group (ECOG) score, and Charlson Comorbidity Index) and oncological characteristics, including tumor subtype (based on the classification of the Skeletal Oncology Research Group (SORG)), new or known cancer, and preoperative neurological status. Potential delays in the chain of care of patients with MESCC were classified into four categories: patient, diagnosis, referral, and surgical delays (Figure 2) [44]. Patient delay refers to the time elapsed from symptom onset to seeking medical attention for MESCC. Diagnostic delay spans the period from initial consultation to accurate diagnosis with MRI and, less commonly, CT myelography. Referral delay is the time elapsed from confirmation of the diagnosis to specialist referral. Surgical delay is the duration from the decision for surgery to its initiation, encompassing preoperative preparations. Interventions to reduce patient and diagnostic delays include patient and physician education on the symptoms and signs of MESCC respectively. These interventions could be used in tandem with a deep learning model for earlier opportunistic diagnosis of MESCC on screening CT scans. Management of referral and surgical delays requires a multidisciplinary approach, comprising the oncologist, radiation oncologist, radiologist, and spine surgeon.

The extraction and de-identification of data from the electronic medical records were performed by a co-researcher who was not involved in the image reviews.

### 2.5. Deep Learning Model Development

Details on the deep learning model (DLM), which utilizes a previously developed and externally validated algorithm [43], are described in the Supplementary Materials.

### 2.6. Statistical Analysis

Inter-rater agreement was assessed using Gwet’s kappa due to the predominance of normal gradings along the entire imaging volumes. Stata software version 17 (StataCorp, College Station, TX, USA) was employed for statistical analysis, with significance set at two-sided *p* < 0.05. Continuous variables were presented as means with standard deviations (SD), while categorical variables were expressed as frequencies and percentages. The strength of agreement for Gwet’s kappa statistics followed the scale proposed by Landis (almost perfect [1–0.81], substantial [0.8–0.61], moderate [0.6–0.41], fair [0.4–0.21], slight [0.2–0], and poor [<0]) [45]. Sensitivities, and specificities were calculated with 95% confidence intervals (CI), and potential reductions in delays to surgical treatment were determined for the four key categories (patient, diagnostic, referral, and surgical delays), and in days from the screening CT to confirmation of high-grade MESCC on MRI.

## 3. Results

### 3.1. Patient Demographics

**Figure 3:**
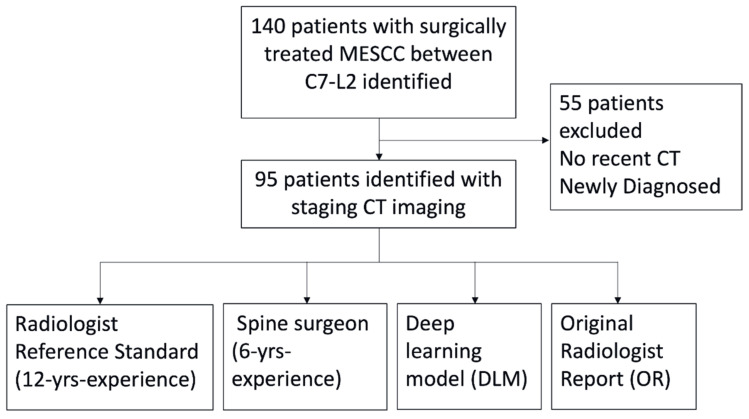
Flow chart of the study design. The DLM was compared against a radiologist (reference standard), spine surgeon, and the original radiology report issued at the time of the CT scan. All CT scans had matching MRI spine scans to confirm the diagnosis of metastatic epidural spinal cord compression (MESCC).

Overall, 95/140 (67.9%) patients with surgically treated MESCC underwent screening CT prior to the MRI (Figure 3). The mean age of the 95 patients was 63 ± 9 (range 40–86) years, with a nearly equal distribution between male (47/95, 49.5%) and female (48/95, 50.5%) participants. The ethnicities of the included patients were Chinese (73/95, 76.8%), Malay (16/95, 16.8%), and Indian (6/95, 6.3%). Most patients had a preoperative ECOG score within the 0–2 range (75/95, 78.9%), while 21.1% (20/95) scored within the 3–4 range. The Charlson Comorbidity Index ranged from zero to 12, with a mean value of eight. Table 1 summarizes the demographic characteristics of the 95/140 patients included in the study.

In terms of the oncological characteristics (highlighted in Table 1), the distribution of tumor growth rates were relatively balanced across the slow (26/95, 27.4%), moderate (34/95, 35.8%), and rapid (35/95, 36.8%) categories, illustrating the heterogeneous nature of MESCC cases. The majority of patients (70/95, 73.7%) entered the study with a known cancer diagnosis, while 26.3% (25/95) received a new diagnosis based on the initial CT findings. The preoperative neurological status presents a spectrum, with 5.3% (5/95) categorized as Frankel A, 52.6% (50/95) as Frankel B or C, and 42.1% (40/95) as Frankel D or E.

### 3.2. Imaging Evaluation for MESCC

Overall, 95/140 (67.8%) patients had undergone preoperative CT imaging. High-grade MESCC was identified in 84/95 (88.4%) of the preoperative CT scans by the reference standard radiologist (J.T.P.D.H.) who had access to the diagnostic MRI for comparison. In contrast, high-grade MESCC was reported in only 32/95 (33.7%) of the preoperative CT scans by the original radiologist (OR).

For the diagnosis of high-grade MESCC on CT, there was almost perfect agreement between the reference standard (J.T.D.P.H.) and the spine surgeon (J.H.J.T.), with a kappa of 0.947 (95% CI = 0.893–1.000) (*p* < 0.001), and between the reference standard (J.T.P.D.H.) and the DLM, with a kappa of 0.891 (95% CI = 0.816–0.967) (*p* < 0.001). There was also almost perfect agreement between the spine surgeon (J.H.J.T.) and the DLM, with a kappa of 0.891 (95% CI = 0.816–0.967) (*p* < 0.001). On the other hand, there was only slight inter-observer agreement between the OR and all other readers, including the DLM (kappa of 0.022, 95% CI = −0.009–0.053), spine surgeon (kappa of 0.125, 95% CI = 0.046–0.204), and the reference standard radiologist (kappa of 0.125, 95% CI = 0.046–0.204) (Table 2).

Compared to the reference standard radiologist, the spine surgeon and DLM showed high sensitivity for the diagnosis of high-grade MESCC on CT, with sensitivities of 97.62% and 100%, respectively. On the other hand, the OR, compared to the reference standard, had a reduced sensitivity of 38.1% (Table 2).

### 3.3. Delays in MESCC Treatment

Analysis of the patient cohort showed that the mean total delay (i.e., from the onset of symptoms, such as back pain, to surgical treatment) was 64 ± 58.0 (range 1–275) days for the 95/140 patients included in the study, who had CT scans performed prior to the diagnostic MRI. On subset analysis, the longest delay was patient delay (i.e., time for the patient to report symptoms to the physician) with a mean of 24 ± 48.7 days (range of 0–271). Diagnostic delay (i.e., time elapsed from first consultation until MRI confirmation of diagnosis) was the second longest delay, with a mean of 15.7 ± 23.3 (range of 0–114) days. Referral and surgical delays contributed the least to total delay, with means of 2.4 ± 4.2 (range of 0–21) days and 4.8 ± 5.7 (range of 0–29) days, respectively.

Our analysis revealed that for those patients with CT studies performed prior to the MRI, there was a mean potential reduction in the total delay by 20 ± 28 (range 1–131) days. A reduction in delay is defined as a shorter period of time elapsed before an accurate diagnosis of the MESCC is made according to the reference standard and the DLM (sensitivity of 100%). A retrospective examination of the electronic medical records showed that 69/95 (72.6%) patients were already symptomatic at the time of the screening CT scan.

## 4. Discussion

Metastatic epidural spinal cord compression (MESCC) poses a significant challenge in cancer care, with delays in diagnosis and treatment being linked to adverse outcomes [46]. Our study aimed to assess the effectiveness of a deep learning model (DLM) in the diagnosis of high-grade MESCC on screening CT scans, and its potential impact on the reduction of diagnostic and treatment delays. Our findings highlight the limitations of relying solely on original radiologist (OR) reports for diagnosing high-grade MESCC. The OR reported high-grade MESCC in only 33.7% (32/95) of the preoperative CT scans, indicating a substantial under-diagnosis. In contrast, our DLM demonstrated almost perfect agreement with both a senior consultant musculoskeletal radiologist and a consultant spinal surgeon, achieving a kappa of 0.891 (95% CI = 0.816–0.967) for both (*p* < 0.001), with the OR showing only slight inter-observer agreement with the DLM and readers (kappa range = 0.022–0.125). This emphasizes the potential of the DLM to enhance diagnostic accuracy and overcome the challenges posed by traditional radiological interpretations.

Our study also addressed the important issue of treatment delays associated with MESCC. The analysis of delay intervals revealed that patient and diagnostic delays contributed significantly to total delay, which has been corroborated by the findings of other studies [44,47,48,49]. Current interventions aimed at mitigating patient and diagnostic delays primarily revolve around educating patients and physicians about MESCC symptoms and signs. The DLM in this study could also play a complementary role in reducing the time to diagnosis, demonstrating a mean potential reduction in total delay of 20 ± 28 (range = 1–131) days. This reduction could be important in enhancing patient outcomes, considering the known association between delayed surgical treatment and increased mortality in MESCC cases. Another potential benefit would be to identify patients with MESCC at an earlier grade, potentially allowing for treatment with primary radiotherapy [50,51] and reducing the need for more invasive surgery.

The value of a DLM lies in its potential to address the under-diagnosis of MESCC on screening CT scans [23,43]. While MRI remains the gold-standard imaging modality for diagnosing MESCC, contrast-enhanced CT scans offer potential for opportunistic screening. Notably, Crocker et al. (2011) reviewed CT scans of 41 patients with suspected MESCC; a sensitivity of 89% and a specificity of 92% were reported for MESCC diagnosis when compared to subsequent MRIs [52]. However, this study focused on patients with symptoms suggestive of MESCC and the reporting radiologists were specifically interrogating the spinal canal to decide on transfer to another center for definitive MRI. More recently, Hallinan et al. (2022) assessed 123 CT scans from 101 patients with known MESCC on MRI and demonstrated that MESCC was only reported in 44.3% of patients by the reporting radiologist [23]. Similar findings were seen in a study by Kim et al. (2023), which assessed a study population of 166 patients with 293 body CT examinations within 30 days of MRI. MESCC was clearly visible in 80.5% (236/293) of body CT examinations but not reported in 65.3% (154/236) of the cases [53].

One primary reason for this under-diagnosis is the tendency for staging or follow-up CT scans to be reported by non-musculoskeletal radiologists, who often concentrate on visceral or nodal metastases, and lack experience in diagnosing MESCC on CT and MRI studies. In the clinical setting, our DLM has the potential to enhance MESCC detection on CT scans by augmenting the reporting radiologists. DLM predictions, represented as bounding boxes, can be overlaid on CT images for radiologist review. This collaborative approach could improve the overall accuracy of MESCC detection and ensure there is oversight of the DLM predictions. Previous applications of DLM in medical imaging, such as lumbar spinal stenosis and chest radiograph evaluation, have demonstrated enhanced accuracy and productivity when used in tandem with radiologists [54,55,56]. However, the successful deployment of DLM in real-world scenarios remains challenging and requires careful consideration of institutional infrastructure and workflow integration [57,58,59].Our study has several limitations. Firstly, the retrospective nature of our investigation introduces biases and the possibility of information gaps. Despite our thorough review of available electronic medical records, patient and oncological data at the time of the CT scans were occasionally incomplete or variable, impacting the overall robustness of our analyses. Secondly, the limited region of coverage, primarily focusing on the thoracic region, poses a constraint. The absence of cervical spine coverage in our screening CT scans (thorax/abdomen/pelvis) restricts the generalizability of our findings to this specific anatomical area. Thirdly, our study focused on a relatively small surgical cohort of patients with MESCC from our institution. To enhance the external validity of our findings, future investigations should utilize a broader patient population from external institutions and consider including patients treated with radiotherapy and/or systemic therapy alone. Lastly, the evaluation of CT scans for MESCC was conducted by an experienced radiologist and spine surgeon. Future work should involve a more diverse set of readers, including in-training radiologists and surgeons who might encounter MESCC diagnoses outside of regular hours. This group of readers could also be augmented by the DLM, which would provide a more realistic assessment of the model’s generalizability and effectiveness in a real clinical setting.

Finally, the low specificity of our model is also a potential limitation. A few factors may have directly or indirectly contributed to this issue, including (as discussed above) the retrospective nature of the data (possible presence of underlying biases), gaps in the radiological and clinical data that were collected for the purpose of this study, and the relatively smaller sample size of our study.

However, we still believe that there exists significant utility for this deep learning model to be incorporated into existing clinical workflows to increase the rate of detection of metastatic epidural spinal cord compression (MESCC). The very high sensitivity of the deep learning model (as demonstrated by the results of our study) allows it to serve as an important screening tool, by drawing attention to potentially suspicious findings on routine staging scans that may otherwise be missed when such scans are interpreted by non-musculoskeletal radiologists. The reviewing radiologist, when alerted to findings of concern, may then quickly review the images to ascertain the presence or absence of MESCC. Hence, the deep learning model, in this regard, functions as a diagnostic adjunct or triage tool (and not a standalone reader), and its implementation could effectively address the problem of under-detection of MESCC by reporting radiologists.

Apart from cancer detection, machine learning has also been utilized for the interpretation of other various non-oncological imaging studies. Relatively lower specificities have been reported for deep learning algorithms used for the detection of consolidation and pneumonia on chest radiographs [60], and for the detection of intracranial hemorrhage (ICH) on brain imaging [61]. Despite these limitations, deep learning techniques and computer-assisted diagnostics (CADs) show significant promise of being adopted as critical diagnostic tools for both of the aforementioned clinical indications. For example, Arbabshirani et al. (2018) described the successful integration of a machine learning model for ICH detection into a clinical practice environment, causing a significant reduction in median time to diagnosis, and allowing for the prompt identification (and, therefore, treatment) of potentially life-threatening cases of ICH on anticoagulation [62].

The above underscores the importance of having an accurate understanding of the capabilities of deep learning models so as to optimize their utilization in actual clinical practice, where their primary purpose should be to complement, rather than replace, human expertise [58]. The development of hybrid diagnostic techniques, which integrate clinical, laboratory and radiological data with deep learning systems [63], increases the dependability of CADs by providing a more holistic view of the diagnostic process. For example, a combined model developed by Zhang et al. (2024), integrating clinical data with deep learning input, was able to outperform all other models in the detection of invasive pulmonary aspergillosis on computed tomography (CT) imaging [64]. Ensemble systems also allow the shortcomings of individual deep learning models to be overcome by a combination of multiple models [65].

Furthermore, with the availability of more cases and external datasets, our deep learning model can be further trained to optimize its specificity. Through further training, the diagnostic thresholds of our deep learning model can be adjusted as appropriate to increase its specificity without significantly sacrificing sensitivity.

## 5. Conclusions

Our study demonstrates the potential of a deep learning model to improve the diagnosis of high-grade MESCC on screening CT scans. The high sensitivity and inter-observer agreement of the DLM compared to the experienced readers indicate the reliability of the DLM. In addition, the potential reduction in mean total delay suggests that integrating the DLM into clinical practice could lead to earlier diagnosis and treatment initiation for MESCC, improving patient outcomes. However, future prospective research is essential to validate these findings and assess the real-world impact of DLM implementation in routine clinical settings.

## Figures and Tables

**Table 1:** Baseline demographic and oncological characteristics of patients with MESCC.

Characteristic	Included Patients (*n* = 95)
Age (years), mean ± SD (range)	63 ± 9 (40–86)
Sex, *n* (%)	
Male	47 (49.5%)
Female	48 (50.5%)
Ethnicity, *n* (%)	
Chinese	73 (76.8%)
Malay	16 (16.8%)
Indian	6 (6.4%)
Preoperative ECOG score, *n* (%)	
0–2	75 (78.9%)
3–4	20 (21.1%)
Charlson Comorbidity Index, mean ± SD (range)	8 ± 2 (0–12)
Tumor subtype (SORG classification), *n* (%)	
Slow growth	26 (27.4%)
Moderate growth	34 (35.8%)
Rapid growth	35 (36.8%)
Diagnosis, *n* (%)	
Known cancer	70 (73.7%)
New diagnosis	25 (26.3%)
Preoperative neurological status (Frankel), *n* (%)	
A	5 (5.3%)
B or C	50 (52.6%)
D or E	40 (42.1%)

Note: ECOG—Eastern Cooperative Oncology Group; SD—standard deviation; SORG—Skeletal Oncology Research Group classification system used to categorize tumor subtypes based on their growth rates.

**Table 2:** Diagnosis of high-grade MESCC on CT for the readers and deep learning model.

Readers *	Kappa (95% CI)	*p*-Value	Sensitivity (95% CI)	Specificity (95% CI)
Spine surgeon (J.H.J.T.)	0.947 (0.893–1.000)	<0.001	97.62 (91.66–99.71)	81.82 (48.22–97.72)
Deep learning model	0.891 (0.816–0.967)	<0.001	100 (95.7–100)	18.18 (2.3–51.8)
Original radiologist report	0.125 (0.046–0.204)	<0.001	38.1 (27.71–49.34)	100 (71.5–100)

* All readers compared against a senior consultant radiologist (J.T.P.D.H.) with access to the diagnostic MRI (reference standard).

## Data Availability

The data presented in this study are not publicly available due to confidentiality and ethical issues. They are available upon request from the corresponding author.

## Supplementary Materials

The following supporting information can be downloaded at https://www.mdpi.com/article/10.3390/cancers17132180/s1, Figure S1: Graphical summary of the deep learning model (DLM) comprising a pipeline for abdominal window axial images with a fixed window width and level set to 400/50 Hounsfield units. References [34,43,66,67] are cited in the Supplementary Materials.

## Abbreviations

The following abbreviations are used in this manuscript:

CI	Confidence interval
CT	Computed tomography
DICOM	Digital imaging and communications in medicine
DL	Deep learning
DLM	Deep learning model
ECOG	Eastern Cooperative Oncology Group
MESCC	Metastatic epidural spinal cord compression
MRI	Magnetic resonance imaging
OR	Original radiologist
PACS	Picture archiving and communication systems
SD	Standard deviation
SORG	Skeletal Oncology Research Group
