# Supplementary material for: Exploring the Potential of a Deep Learning Model for Early CT Detection of High-Grade Metastatic Epidural Spinal Cord Compression and Its Impact on Treatment Delays
Source: Cancers (Basel). 2025 Jun 28;17(13):2180. doi: 10.3390/cancers17132180 (PMC12248473; doi:10.3390/cancers17132180)
Supplement: Supplementary file 1 [file cancers-17-02180-s001.zip › cancers-3694746-supplementary.pdf]

Supplementary Materials

Deep Learning Model Development

This study employed a deep learning pipeline with a consecutive region of interest (ROI) detector followed by a classification/grading system. This pipeline was initially developed by Hallinan and Zhu et al. (2022) [34] and fine-tuned by the same group utilizing stochastic gradient descent (SGD) for optimization [43]. The initial phase involved constructing a Faster R-CNN combined with ResNet50 as its backbone network architecture for ROI detection. Following ROI detection, a combined window learning (CWL) method with average fusion was implemented for ROI classification. The CWL model, utilizing ResNeXt50 as its backbone, incorporated window-specific batch normalization layers to capture unique information from CT scans with different window characteristics. The deep learning algorithm was optimized using a separate validation dataset, and the final prediction probability was derived by averaging predictions from each window. In this study the abdominal window images were used alone.

Inference and Model Optimization

During inference, the model processed input with multi-window information, outputting the average prediction probability. The deep learning pipeline, developed on the Apache SINGA platform, utilized MLCask for efficient management of algorithm versions [66,67]. The study's deep learning algorithms, with a batch size of 72 and inputs from three different windows, were implemented on a NVIDIA GeForce GTX 1080p GPU. Code for the deep learning algorithms is accessible at https://github.com/NUHS-NUS-SpineAI/SpineAI-Bilsky-Grading-CT. Notably, the prior optimization study included an ablation study to assess performance benefits arising from the framework and advanced backbone. The study's methodology forms a comprehensive pipeline, depicted graphically in **Figure S1**, highlighting the sequential processes involved in ROI detection and classification for normal/low versus high-grade MESCC.

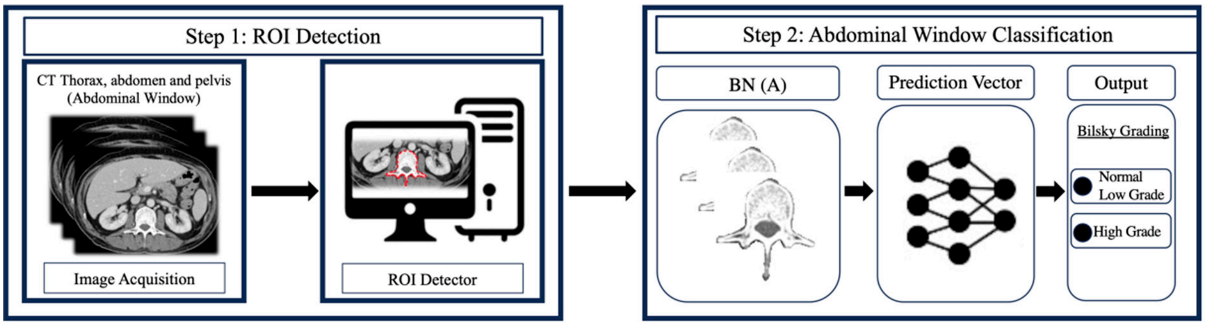

Figure S1. Graphical summary of the deep learning model (DLM) comprising a pipeline for abdominal window axial images with a fixed window width and level set to 400/50 Hounsfield units. The deep learning pipeline processes input data (axial images) from CT scans of the thorax, abdomen, and pelvis. In the initial stage, a region of interest (ROI) detector is employed to identify ROIs across all images. Subsequently, a ROI classifier with batch normalization layers is applied to individual input images to generate prediction vectors. These prediction vectors are then element-wise added and averaged to form the final prediction output. BN denotes Batch Normalization.

References

34. Hallinan, J.; Zhu, L.; Zhang, W.; Kuah, T.; Lim, D.S.W.; Low, X.Z.; Cheng, A.J.L.; Eide, S.E.; Ong, H.Y.; Muhamat Nor, F.E.; et al. Deep Learning Model for Grading Metastatic Epidural Spinal Cord Compression on Staging CT. *Cancers* **2022**, *14*, 3219. https://doi.org/10.3390/cancers14133219
43. Hallinan, J.; Zhu, L.; Zhang, W.; Ge, S.; Muhamat Nor, F.E.; Ong, H.Y.; Eide, S.E.; Cheng, A.J.L.; Kuah, T.; Lim, D.S.W.; et al. Deep learning assessment compared to radiologist reporting for metastatic spinal cord compression on CT. *Front. Oncol.* **2023**, *13*, 1151073. https://doi.org/10.3389/fonc.2023.1151073
66. Ooi, B.C.; Tan, K.-L.; Wang, S.; Wang, W.; Cai, Q.; Chen, G.; Gao, J.; Luo, Z.; Tung, A.K.H.; Wang, Y.; et al. SINGA: A Distributed Deep Learning Platform. In Proceedings of the 23rd ACM international conference on Multimedia, Brisbane, Australia, 26–30 October 2015; Association for Computing Machinery: New York, NY, USA, pp. 685–688.
67. Luo, Z.; Yeung, S.H.; Zhang, M.; Zheng, K.; Zhu, L.; Chen, G.; Fan, F.; Lin, Q.; Ngiam, K.Y.; Ooi, B.C. MLCask: Efficient Management of Component Evolution in Collaborative Data Analytics Pipelines. In Proceedings of the 2021 IEEE 37th International Conference on Data Engineering (ICDE), Chania, Greece, 19–22 April 2021
